# Promoting Problem-Solving Among Low-Income Adults With Type 2 Diabetes: Cluster-Randomized Controlled Trial of a Mobile Health Intervention With SMS Text Messaging (Mobile Diabetes Detective)

**DOI:** 10.2196/82305

**Published:** 2026-07-13

**Authors:** Lena Mamykina, Arlene M Smaldone, Suzanne R Bakken, Heather Cole-Lewis, Elizabeth M Heitkemper, Haomiao Jia, Rita Kukafka, Jonathan N Tobin, Andrea Cassells, Patricia G Davidson, Elizabeth D Mynatt, George Hripcsak

**Affiliations:** 1 Columbia University New York, NY United States; 2 University of Texas Austin Austin, TX United States; 3 Clinical Directors Network New York, NY United States; 4 The Rockefeller University New York, NY United States; 5 West Chester University West Chester, PA United States; 6 Northeastern University Boston, MA United States

**Keywords:** mHealth, intervention, self-management, problem solving, randomized clinical trial, practice-based research, primary care

## Abstract

**Background:**

Problem-solving is essential for the self-management of type 2 diabetes but remains challenging for underserved individuals. Although mobile health (mHealth) interventions can improve diabetes self-management, few focus on problem-solving.

**Objective:**

This study evaluates the efficacy of Mobile Diabetes Detective (MoDD), a fully automated web-based intervention with SMS text messaging that provides problem-solving support tailored to self-monitoring data, for improving glycemic control among medically underserved adults with type 2 diabetes.

**Methods:**

This open-label, 1:1 cluster-randomized controlled trial was conducted in 2013-2018. Participants were adults with type 2 diabetes (glycated hemoglobin [HbA1c] >7.5%) receiving care at 8 Federally Qualified Health Centers serving medically underserved communities in the New York metropolitan area. The centers served as clusters and were randomized using computer-generated allocation. Recruitment and study sessions were conducted either in person or in a hybrid format. The intervention arm used MoDD for 12 months, whereas the control arm received standard diabetes education and routine care. The primary outcome was the change in HbA1c from baseline to 12 months, recorded from medical chart data. We hypothesized greater improvement in the intervention arm than in the control arm. Secondary outcomes included psychosocial measures. Outcomes were compared between groups using intention-to-treat analyses. This report presents the final analysis of the outcomes.

**Results:**

This trial randomized 248 participants (intervention arm: n=126; control arm: n=122); 219 were included in the final analysis (intervention arm: n=111; control arm: n=108). Participants were predominantly female (147/219, 67.1%) and ethnically and racially diverse (112/219, 51.1%, Hispanic and 92/219, 42%, African American), with a mean baseline HbA1c of 9.9%. Overall, of the 111 participants, 44 (39.6%) engaged with MoDD at least once weekly in the first 30 days, and 22 (19.8%) engaged at least once weekly in the first 90 days. HbA1c did not differ significantly between groups at baseline (intervention: 9.81%, 95% CI 9.42%-10.20%; control: 9.95%, 95% CI 9.55%-10.34%; difference=0.14%, P=.63) or at 12 months (intervention: 9.36%, 95% CI 8.95%-9.78%; control: 9.58%, 95% CI 9.15%-10.01%; difference=–0.22%, P=.47). Both groups demonstrated reductions in HbA1c from baseline to 3 months. Sustained within-group improvement at 12 months was observed in the intervention group but not in the control group. No intervention-related adverse events were reported.

**Conclusions:**

This study evaluated the impact of a mobile intervention for problem-solving in diabetes. MoDD is innovative because it operates autonomously and tailors support to individuals’ self-monitoring data. Although there was no significant between-group difference in HbA1c, the intervention group showed sustained within-group improvement at 12 months. These findings highlight the potential long-term benefits of autonomous mHealth interventions for problem-solving. The study observed an increase in diabetes distress, possibly reflecting heightened awareness of uncontrolled blood glucose levels. If implemented in clinical practice, MoDD could complement diabetes education and help improve glycemic control.

**Trial Registration:**

ClinicalTrials.gov NCT02021591; https://clinicaltrials.gov/ct2/show/NCT02021591

## Introduction

### Scientific Background and Rationale

Well-developed problem-solving is essential to the successful management of type 2 diabetes [1-3], results in better diabetes self-care [4-7], and leads to improvements in clinical outcomes [8-10]. Problem-solving is defined as the process of translating self-care techniques into self-management behaviors and the ability to implement effective solutions to overcome personal, environmental, social, and knowledge-based barriers to self-management [11]. Problem-solving is central to many self-management and behavior change programs [12-15]. The Association of Diabetes Care and Education Specialists includes problem-solving as a critical self-care behavior [16]. However, developing problem-solving skills presents a considerable challenge [17]. Interventions that promote problem-solving often rely on health care professionals to deliver problem-solving education and training in individual or group settings [9,10,18-24]. Although these interventions have demonstrated benefits, they are resource-intensive and often inaccessible to individuals living in medically underserved, low-resource communities with limited access to diabetes self-management education [25].

### Prior Work in Mobile Health for Problem-Solving in Diabetes

With over 97% of Americans owning a cell phone [26], and cell phone ownership rates even higher worldwide and among members of ethnic and racial minority groups [27], mobile health (mHealth) and SMS text messaging interventions can reach broader and more diverse populations [28-30]. Past research has established the efficacy of mHealth interventions for diabetes self-management [31-33], although smaller effect sizes have been reported among minority populations [34]. However, only a few mHealth interventions proposed thus far specifically focus on promoting problem-solving [35-37]. For example, recent systematic reviews of mHealth interventions for diabetes self-management concluded that current technologies often support healthy eating, monitoring, medication adherence, and physical activity but lag far behind in supporting problem-solving, healthy coping, and risk reduction [35,36]. Another review suggested that mHealth interventions for diabetes self-management focus more on behavior change than on cognitive skills such as problem-solving [37]. Furthermore, the mHealth technologies that did focus on problem-solving either relied on health care professionals to deliver problem-solving education, thereby limiting scalability [38], or provided such education in simulated, sometimes game-like settings rather than supporting problem-solving around individuals’ actual glycemic challenges identified through self-monitoring data [23,24,39,40]. As a result, there are clear gaps in the availability of scalable and personalized mHealth interventions focused on fostering problem-solving skills in diabetes.

### Problem and the Type of Solution

Mobile Diabetes Detective (MoDD) is theoretically grounded in a problem-solving framework developed by D’Zurilla and Goldfried [41], informed by our prior research on diabetes self-management [42-44], and developed using a user-centered design approach [45]. It was designed to complement, rather than replace, standard diabetes education and is introduced after individuals have completed basic diabetes education. In contrast to prior interventions for problem-solving in diabetes, MoDD offers a 2-fold innovation. First, MoDD is fully automated and does not rely on the involvement of health care professionals. Second, it uses individuals’ self-monitoring data to tailor problem-solving support to each individual’s glycemic challenges. We evaluated MoDD in a cluster-randomized, nonblinded clinical trial involving medically underserved individuals with type 2 diabetes.

### Specific Objectives and Hypotheses

The specific objective of this study was to address gaps in current research and evaluate the efficacy of a novel mHealth intervention, MoDD, for facilitating problem-solving and self-care behaviors in diabetes self-management [46]. The primary hypothesis was that exposure to MoDD for 12 months (intervention arm) would result in greater improvement in individuals’ glycated hemoglobin (HbA_1c_), the primary outcome, from baseline to 12 months compared with standard diabetes education (control arm).

## Methods

### Study Design and Registration

This study was a 2-arm, parallel, nonblinded cluster-randomized controlled trial in which Federally Qualified Health Centers (FQHCs) served as clusters and were randomized in a 1:1 ratio to intervention or control conditions. The control arm received standard diabetes education and care, whereas the intervention arm received standard diabetes education and care in addition to using MoDD for up to 12 months. The trial was conducted in economically disadvantaged communities in the New York metropolitan area. The primary outcome was the difference in HbA_1c_ between study arms at 12 months after baseline. The study was conducted between 2013 and 2018. The study was prospectively registered with ClinicalTrials.gov before enrollment of the first participant (Trial Registration: NCT02021591). The study was first posted on ClinicalTrials.gov on December 18, 2013, and the first participant was enrolled on December 30, 2013. The trial protocol and statistical analysis plan are available through the trial registry.

### Trial Settings

Eight FQHCs in the New York metropolitan area that are members of the Clinical Directors Network (CDN), a primary care practice-based research network, were recruited to participate in this study. All FQHCs provided care to patients from low-resource communities, with a high proportion of patients either insured through Medicaid or uninsured. The population served by each FQHC reflected the characteristics of its community in terms of race, ethnicity, and preferred language. FQHCs varied in the type of diabetes education provided and in the availability of an endocrinologist on-site. To reduce the potential impact of differing clinical practices on study outcomes, the inclusion criteria for FQHCs were as follows: (1) operational for 2 or more years; (2) provide primary care to 5000 or more adult patients annually; (3) have an established diabetes self-management education and support (DSMES) program; (4) have 2 or more staff members who provide diabetes education on-site; and (5) previous participation in at least one Bureau of Primary Health Care–sponsored Health Disparities Collaborative project [47].

### Eligibility Criteria for Participants

Participants met the following eligibility criteria: (1) age 18-65 years; (2) diagnosis of type 2 diabetes with HbA_1c_ ≥7.5%; (3) patient of the health center for at least 6 months; (4) participation in at least one diabetes education session (group or individual) at the study site within the previous 6 months; (5) proficiency in either English or Spanish; and (6) ownership of a basic or more advanced cell phone. The exclusion criteria were as follows: (1) pregnancy; (2) presence of a serious illness (eg, cancer requiring active treatment); (3) presence of cognitive impairment; and (4) plans to leave the FQHC within the next 12 months. Further details regarding participants and recruitment are available elsewhere [48]. The study did not explicitly include computer or internet literacy as an eligibility criterion; instead, ownership of a mobile phone was considered to imply basic proficiency in using mobile technology. No minimum level of digital literacy was required, and participants received training to ensure they could access the intervention.

### Intervention

#### Theoretical Framework

**Figure 1:**
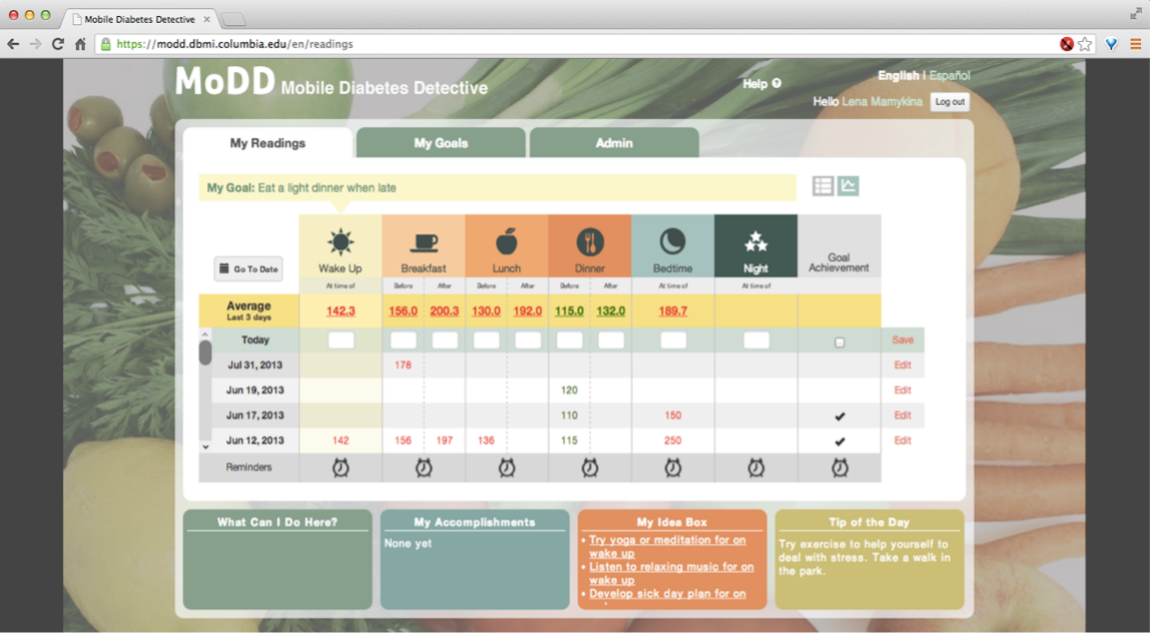
User dashboard of Mobile Diabetes Detective. The view shown is the My Readings tab, where an individual’s blood glucose readings are organized into patterns (eg, wake-up, before and after breakfast/lunch/dinner, and nighttime), with readings above recommended levels highlighted in red. Other tabs include My Goals, where individuals can review their goals and track progress toward achieving them, and Admin, where account information can be reviewed.

MoDD is a fully automated mHealth intervention designed for independent use by individuals with type 2 diabetes. Because of its focus on problem-solving, the design of MoDD was grounded in a problem-solving framework rather than in behavior change theories. Previous studies have provided ample evidence supporting the association between improved problem-solving and improved self-management, thereby justifying the choice of a more focused and narrower framework [6]. We reviewed existing frameworks for problem-solving in diabetes (eg, [1,9,41]) and selected the framework developed by D’Zurilla and Goldfried [41] as the most appropriate because of its specific focus on the problem-solving process. Specifically, the framework outlines 5 stages of problem-solving: (1) general orientation or “set,” (2) problem definition and formulation, (3) generation of alternatives, (4) decision-making, and (5) verification. Accordingly, MoDD includes features designed to support each of these stages. Table 1 outlines the basic components of the MoDD design and their mapping to the stages of the problem-solving framework. Figure 1 presents screenshots of the MoDD user dashboard.

**Table 1:** Stages of the D’Zurilla and Goldfried [41] problem-solving framework and corresponding MoDD^a^ components.

Problem-solving stage	MoDD main components
General orientation	Track BG^b^ levels (at waking, before and after each meal, and at bedtime) for at least 3 days and report them to MoDD via dashboard or SMS text messages. MoDD identifies patterns with consistent deviations from ranges recommended by health care providers (70-130 mg/dL for fasting BG, and 70-180 mg/dL for after-meal BG) and highlights them as problematic *glycemic control patterns*.
Problem definition and formulation	Select *a glycemic control pattern* for improvement (eg, “High blood glucose after breakfast”), review *potential behavioral triggers*—behaviors that may be contributing to the selected pattern (eg, “I rarely include protein in my breakfast”), and select a trigger that is most consistent with an individual’s own behaviors. The list of triggers is provided in the MoDD knowledge base [45].
Generation of alternatives	Review a list of alternative evidence-based behaviors that could address the underlying glycemic problem (eg, for the selected trigger “I rarely include protein in my breakfast,” select an alternative behavior to “Include a tablespoon of peanut butter or a boiled egg with breakfast”).
Decision-making	Set an *action-oriented goal* consistent with the chosen alternative behavior and receive daily goal reminders.
Verification	Receive *tailored feedback via SMS text messages* about progress toward achievement of target BG ranges.

^a^MoDD: Mobile Diabetes Detective.

^b^BG: blood glucose.

MoDD uses an extensive knowledge base designed and validated in collaboration with practicing diabetes educators and individuals with type 2 diabetes [45]. The knowledge base includes a comprehensive list of behavioral triggers and evidence-based, action-oriented goals, each accompanied by a brief educational segment explaining how specific behaviors may lead to changes in blood glucose (BG) levels. These educational segments are tailored to participants’ health literacy levels, which were assessed during MoDD registration using a nutritional knowledge in diabetes scale [49]. Furthermore, the knowledge base includes motivational messages tailored to participants’ readiness to change dietary and physical activity behaviors [50], which were also assessed during MoDD registration.

MoDD is a fully automated intervention and does not involve human support. Participants received automated daily SMS text messages reminding them of their selected goals and prompting them to track their BG levels. MoDD was designed to enhance, rather than replace, standard diabetes education by placing greater emphasis on problem-solving without relying on the involvement of health care professionals. Further details regarding the design of MoDD are described elsewhere [51].

#### Patient and Public Involvement

MoDD was developed using an iterative, user-centered design process involving individuals with type 2 diabetes recruited from FQHCs that were not participating in the trial [51]. MoDD was developed by a team of software developers who served as consultants on this research project and were not involved in the collection or analysis of data related to study outcomes. MoDD was available in both English and Spanish. The MoDD website [52] includes screenshots and demonstrations of MoDD functions and features for digital preservation.

#### Delivery, Access, and Training

Intervention dose was operationalized as the number of MoDD logins and the number of BG readings recorded within the platform. Participants were encouraged to use MoDD to track their BG up to 8 times per day (upon waking, before and after each meal, and at bedtime) for at least 3 days and to log in at least weekly to monitor changes in BG patterns throughout the remainder of the study period.

Research staff instructed participants in the intervention arm to use MoDD independently through either its SMS text messaging or web app features for a minimum of 4 weeks and up to the final follow-up visit at 12 months. All participants in the intervention arm received a 1-hour training session on the use of the intervention. Participants accessed MoDD through a secure web portal [52] using individual login credentials.

The MoDD intervention content, screenshots, and example SMS text messages are provided in Multimedia Appendix 1 to support replicability. The version of the system used in this trial remained unchanged throughout the study period. No modifications were made to MoDD after trial commencement, and there were no notable bug fixes, downtime events, or content changes during the study. The intervention platform used in the trial remains archived for replication purposes.

### Trial Design

The study was conducted between 2013 and 2018. To prevent cross-contamination among patients within the same center, the study used a cluster-randomized, nonblinded clinical trial design in which participating FQHCs were randomized to either the intervention arm (n=4) or the control arm (n=4). Because of differences in the delivery of DSMES across FQHCs, study sites were stratified as follows: (1) DSMES delivered through group classes with certified diabetes educators on-site; and (2) DSMES delivered through individual consultations with no certified diabetes educators on-site. The FQHCs were then randomized in a 1:1 ratio to the intervention and control arms within each stratum. There were no changes to the study methods or protocol after trial commencement.

### Recruitment and Informed Consent Procedures

Participants were recruited through closed recruitment methods, including referrals from FQHC clinicians and staff, direct mailings, posted flyers, and face-to-face recruitment by approaching adults with a type 2 diabetes mellitus diagnosis who had checked in and were waiting for their appointments.

During the in-person consent visit, participants were informed that MoDD is a mobile intervention designed to help them solve specific problems related to diabetes management. They were also informed that they would be asked to check their BG levels several times per day for at least the first 3 days of the study and at least weekly for the remainder of the study period.

### Study Procedures

Patients recruited from intervention-arm sites received access to the MoDD app immediately after baseline assessment and intervention training. The intensive intervention phase lasted 4 weeks, followed by a maintenance phase during which participants continued to access MoDD for up to 12 months. At the end of the study period (12 months) and after completion of the final follow-up assessments, patients recruited from control-arm sites were provided access to the MoDD app along with instructions for its use. As randomization occurred at the cluster (FQHC) level, no additional blinding after randomization was possible. Research staff enrolled participants and assigned them, within their corresponding clusters, to the appropriate study arm.

The study protocol included 5 study visits: (T1) consent/baseline visit; (T2) training visit; (T3) 4 weeks, marking the end of the main intervention period; (T4) 3 months; and (T5) 12 months. Immediately following the T1 visit, participants in the intervention arm attended the training visit (T2). Participants in the usual care arm did not receive any training on MoDD and continued to receive standard diabetes care. During the T2 visit, participants in both arms were provided with a voucher to purchase test strips for their BG meters (2 strips per day for the 4-week main intervention period) and US $10 in cash to offset costs associated with SMS text messaging and data use during the study. All participants received a diabetes education brochure developed by the New York City Department of Health and Mental Hygiene. During sessions T3-T5, participants completed validated surveys administered in the same mode used in the original validation studies (ie, self-administered on paper or administered by staff by phone or in person, based on participants’ preferences), rather than through online questionnaires. If a participant reported distress during the assessments, they were referred to their primary care provider for follow-up care.

Research staff instructed participants in the intervention arm to use MoDD independently through either its SMS text messaging or its web app features and to log in at least weekly for a minimum of 4 weeks and until the final follow-up visit (12 months); no BG test strips or incentives were provided after the initial 4 weeks. To ensure that all participants had access to the MoDD web app, the FQHCs were equipped with dedicated laptops that participants in both study arms could use to access MoDD and receive additional training. Notably, with the exception of the training visit, all other study procedures (eg, compensation for study visits, BG monitoring test strips, data plans, and access to on-site computers) were identical across study arms. One week after training, a member of the research team conducted a follow-up phone call with each participant to answer questions and encourage MoDD use. Participants were also encouraged to contact study coordinators via SMS text message or phone if they required additional assistance with MoDD features. Further details regarding the trial design are available elsewhere [48].

Upon completion of the study, participants were invited to participate in qualitative interviews to discuss their experiences with MoDD. Further details regarding the qualitative study are available elsewhere [51].

Recruitment was conducted from December 2013 to October 2015. Follow-up assessments were conducted from January 2014 to February 2017.

### Safety and Security Procedures

To ensure participant safety, the research team met weekly to review enrollment and recruitment reports. In addition, members of the research team monitored participants’ engagement with MoDD through its interactive dashboard. The study was also overseen by a Data and Safety Monitoring Board, which met annually to review study procedures and ensure participant safety and data security. All data transmitted through MoDD were encrypted using secure HTTPS protocols and stored on password-protected institutional servers.

### Randomization, Blinding, and Random Allocation Sequence

Cluster randomization of FQHCs into study arms was performed using a random-number sequence generated by an off-site study statistician. The statistician generated the allocation sequence using a random-number generator, and participating FQHCs were allocated to study conditions within their respective strata. Clusters were randomized before participant recruitment; therefore, participants were recruited with knowledge of their clinic’s allocation. Care providers were assigned to trial groups according to their FQHC. The statistician had no involvement in recruitment or data collection, and the allocation sequence was concealed from research staff until clusters had been assigned. Due to the cluster-randomized design and the nature of the intervention, participants, clinicians, and research staff were not blinded to study assignment. Study personnel recruited participants and assigned them to the study condition corresponding to their cluster (FQHC) randomization. Participants assigned to the control arm were offered the opportunity to engage with MoDD after completion of the 12-month main study period. To minimize selection bias, all eligible participants from each FQHC were recruited until the target sample size was achieved.

### Primary and Secondary Outcomes

The primary outcome was HbA_1c_ extracted from participants’ electronic medical records, with 12 months after baseline designated as the primary end point. A 1% decrease in HbA_1c_ is associated with a significant reduction in the rate of diabetes complications, thereby establishing a clinically meaningful effect-size target [53]. Secondary outcomes included individuals’ problem-solving abilities (Diabetes Problem-Solving Inventory [DPSI]) [46], diabetes self-care behaviors (Summary of Diabetes Self-Care Activities Questionnaire [SDSCA]) [54], diabetes self-efficacy (Diabetes Self-Efficacy Scale [DSES]) [55], and diabetes distress (Problem Areas in Diabetes [PAID]) [56]. The DPSI was administered by study personnel who received at least 10 hours of training and participated in 5 synchronization sessions to ensure consistency. The study included only validated surveys, which were administered in the same mode used in their original validation studies (ie, not through online questionnaires). For engagement assessment, engagement was defined as the frequency of logins per day. To assess participants’ qualitative impressions, a qualitative study was conducted concurrently with the clinical trial. This qualitative study was completed before the end of the trial, and its findings were published in a separate manuscript [51]. There were no changes to the study outcomes after trial commencement.

We hypothesized that exposure to MoDD would result in the following:

- H1 (Primary): A greater improvement in individuals’ HbA1c from baseline to 12 months in the intervention arm compared with the control arm.
- H2 (Secondary): A greater improvement in individuals’ problem-solving abilities (DPSI), self-care behaviors (SDSCA), self-efficacy (DSES), and diabetes distress (PAID) from baseline to 12 months in the intervention arm compared with the control arm.

### Sample Size Determination

We were guided by published studies in establishing anticipated effect sizes [29]. The intracluster correlation coefficient (ICC) estimate of 0.02 was derived from previous practice-based intervention trials conducted in FQHC settings. Assuming 90% power, a 2-tailed α level of .05, and an ICC of 0.02, we estimated that 18 participants per FQHC would be required, resulting in a total sample size of 144 participants to achieve sufficient statistical power. Anticipating up to 30% attrition at the 12-month follow-up, we increased the recruitment target to 200 participants.

### Statistical Methods

The primary outcome in this study was improvement in HbA_1c_, the primary outcome measure, from baseline to 12 months. Additional outcomes included improvement in problem-solving, self-efficacy, diabetes self-care behaviors, and diabetes distress. For this intent-to-treat analysis, we used an individual growth model, a special case of linear mixed models, to estimate HbA_1c_ scores at baseline, 4 weeks, 3 months, and 12 months and to compare these estimates across time points within each study arm and between the intervention and control arms. The model included a site-specific random intercept to account for different baseline scores across participating sites and repeated-measures data. The dependent variable was HbA_1c_. The independent variables were study arm (intervention vs control) and period (baseline, 4 weeks, 3 months, and 12 months, with 12 months designated as the primary study end point). An interaction term between study arm and period was included to compare outcomes between the 2 study arms. Similar models were used for each secondary outcome, including problem-solving (DPSI), diabetes self-care behaviors (SDSCA), diabetes distress (PAID), and diabetes self-efficacy (DSES). We used a critical value of *P*=.02 (=0.05/3; Bonferroni correction) to adjust for multiple comparisons across the 3 follow-up time points (4 weeks, 3 months, and 12 months relative to baseline). For the 5 SDSCA subscales, false discovery rate–adjusted *P* values were calculated. Similar models were also used to examine longitudinal changes within each study arm.

A similar linear mixed model was used to examine the association between MoDD use and changes in the primary outcome (HbA_1c_) within the intervention arm. Usage was measured by the number of MoDD logins and the total number of BG readings entered into MoDD. As both variables were skewed, log transformations were applied. Engagement metrics were analyzed to explore dose-response relationships between platform use and glycemic outcomes.

Notably, we did not examine trends in HbA_1c_ from baseline to 12 months across engagement strata because of data limitations, including a large proportion of missing values and a small sample size. Furthermore, to examine the potential association between distress and engagement, we included diabetes distress (PAID) in the model as a confounder. The difference between the adjusted and unadjusted estimates was small (see Multimedia Appendix 1). Finally, we conducted a sensitivity analysis by excluding observations from the intervention arm with 0 engagement. We then estimated changes in HbA_1c_ across the 2 study arms. No differences in the estimates were observed, likely because there were very few observations in the intervention arm with 0 engagement.

The empirical intracluster correlation coefficient (ICC) observed was 0.016. For fixed-effect estimation, we used the default containment method for fixed-effect models in the SAS PROC MIXED procedure to calculate denominator degrees of freedom. No additional analyses, such as subgroup or adjusted analyses, were conducted.

### Analysis of Missing Values

We calculated the percentage of missing values by period and across selected baseline variables. There was no clear evidence of bias in missingness according to the selected baseline variables (see Multimedia Appendix 1). The Missing Completely at Random test [57] indicated that the data were missing completely at random (*P*=.09). Furthermore, we used logistic regression to examine the association between missing values for the primary outcome (HbA_1c_) and 5 baseline variables (intervention arm, age, gender, education, and baseline 2-item Patient Health Questionnaire) within the same period. No significant association was found (*P*=.67) between missingness and any of the baseline variables. We also estimated the primary outcome (HbA_1c_) using 2 methods for handling missing data: full-information maximum likelihood and multiple imputation. The standardized mean differences (SMDs) between the full-information maximum likelihood and current methods were less than 0.13 for all estimates. The SMDs between the multiple imputation and current methods were all less than 0.5. According to Cohen [58], SMD values of 0.2-0.5 are considered small, values of 0.5-0.8 are considered medium, and values over 0.8 are considered large. Therefore, the impact of missing data on bias in the intervention effect estimates was small, supporting the robustness of the findings presented below. A full analysis of missing data is provided in Multimedia Appendix 1.

### Ethical Considerations

The study was approved by the Institutional Review Boards of Columbia University Medical Center, CDN, and Family Health Centers at NYU, and was prospectively registered on ClinicalTrials.gov (Protocol ID NCT02021591). All participants provided written informed consent in either Spanish or English before participation (the consent form is included in Multimedia Appendix 1).

Participants’ confidentiality was protected by assigning identification codes to all study records, and all study data were stored in secure, password-protected databases on secure servers at CDN and Columbia University. Access to study records was restricted to research staff only.

Participants received up to US $130 to compensate for travel expenses, SMS text messaging and cellular data use, time, and effort, distributed as follows: US $15 after the baseline visit, US $15 after the second visit, US $25 after the third visit, US $30 after the fourth visit, US $35 after the fifth visit, and an additional US $10 to cover the cost of SMS text messaging during the 4-week intervention period. This paper and Multimedia Appendix 1 do not include any images that identify individual participants or users.

### Harms

To minimize potential harm, MoDD monitored BG readings submitted by participants during the study. If reported readings fell outside the thresholds established by each participating FQHC, MoDD activated a safety protocol defined by the respective FQHC. For example, participants received an SMS text message alerting them to critical glucose levels and advising them to contact their health care provider, with contact information provided in the message.

This study is reported in accordance with the CONSORT (Consolidated Standards of Reporting Trials; Multimedia Appendix 2) 2025 statement and the CONSORT-EHEALTH extension (Multimedia Appendix 3) for reporting digital health interventions [59].

## Results

### Participant Randomization, Allocation to Intended Treatment, and Inclusion in Analysis

**Figure 2:**
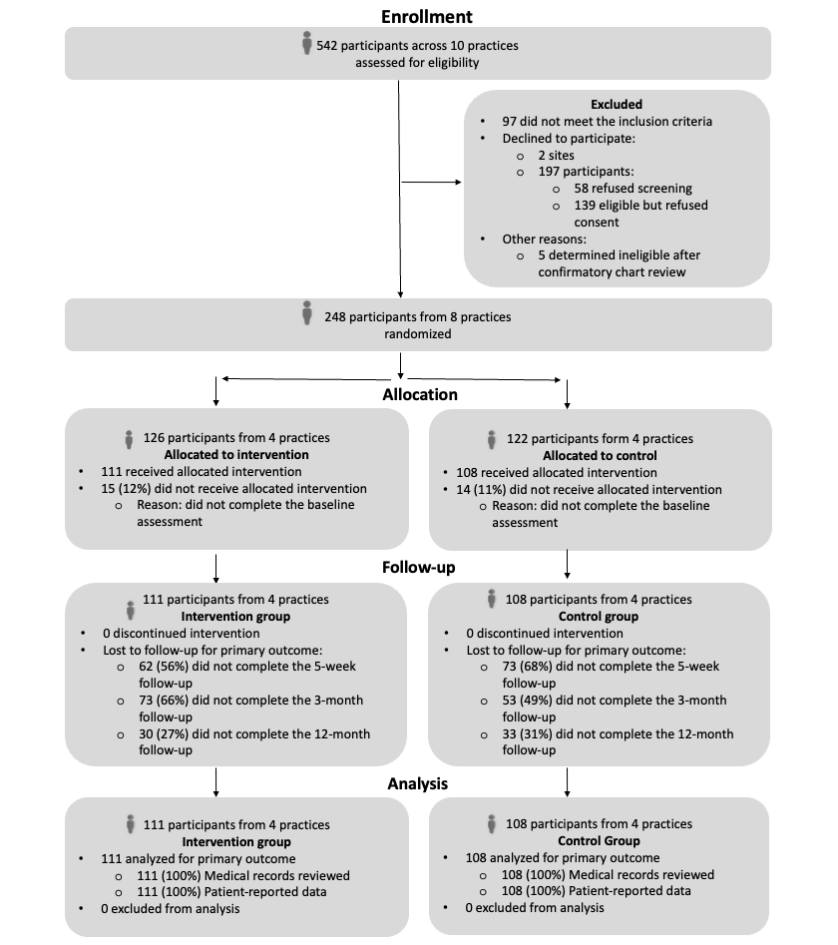
CONSORT (Consolidated Standards of Reporting Trials) flow diagram for the cluster-randomized clinical trial of Mobile Diabetes Detective (MoDD), a mobile health intervention designed to facilitate problem-solving in the self-management of type 2 diabetes. Participants in the control arm received standard diabetes education and care, whereas those in the intervention arm additionally used MoDD for up to 1 year. The trial was conducted between 2013 and 2018 among economically disadvantaged communities in the New York metropolitan area. The diagram summarizes participant-level recruitment, allocation, follow-up, and retention.

Given the cluster-randomized design of this trial, recruitment and retention are reported at both the cluster and participant levels (Figure 2). Ten FQHCs were assessed for eligibility, and 8 were recruited to participate in the trial (2 sites declined participation). These 8 sites were randomized to the intervention arm (n=4; median number of participants per site=31, range 30-34) or the control arm (n=4; median number of participants per site=30.5, range 30-31). No sites were lost to follow-up.

A total of 542 adult patients were identified as potentially eligible for study participation and approached for recruitment. Of these 542 individuals, 97 (17.9%) did not meet the eligibility criteria (most commonly because of a lack of a diabetes diagnosis), 197 (36.3%) declined participation, and 248 (45.8%) consented to participate in the study. Following additional chart review, 5 individuals were determined to be ineligible and were excluded from the study. The remaining 248 participants were randomized to the 2 study conditions according to their corresponding FQHC: 126 to the intervention arm (MoDD) and 122 to the control arm (usual care). Of the 248 randomized participants, 219 (88.3%) completed the baseline assessment and received the allocated intervention (111 in the intervention arm and 108 in the control arm).

HbA_1c_ values measured as part of usual care were extracted from participants’ electronic health records; therefore, HbA_1c_ values were unavailable for participants who missed their regularly scheduled provider visits. For this intent-to-treat analysis, we included all participants with HbA_1c_ values aligned with study milestones, regardless of their attendance at study visits. HbA_1c_ values were unavailable for the following participants: at baseline, data were available for all participants in both study arms; at the 4-week follow-up, data were unavailable for 62 of 111 (55.9%) participants in the intervention arm and 73 of 108 (67.6%) participants in the control arm; at the 3-month follow-up, data were unavailable for 73 (65.8%) participants in the intervention arm and 53 (49.1%) participants in the control arm; and at the 12-month follow-up, data were unavailable for 30 (27%) participants in the intervention arm and 33 (30.6%) participants in the control arm (see Figure 2). The primary reason for missing HbA_1c_ values was the absence of routine clinical visits during the study period.

Survey-based outcome measures were collected during study visits. For these outcomes, the following participants did not complete assessments at the scheduled time points: at baseline, 31 of 111 (27.9%) participants in the intervention arm and 21 of 108 (19.4%) participants in the control arm; at the 3-month follow-up, 23 of 111 (20.7%) participants in the intervention arm and 19 of 108 (17.6%) participants in the control arm; and at the 12-month follow-up, 24 of 111 (21.6%) participants in the intervention arm and 13 of 108 (12%) participants in the control arm.

Of the 248 adults enrolled in the study, 219 were included in the final intent-to-treat analysis (111 in the intervention arm and 108 in the control arm). As randomization occurred at the cluster level before participant recruitment, participants who did not complete the baseline assessment after randomization (n=15 in the intervention arm and n=14 in the control arm) were excluded from the intent-to-treat analysis because of missing baseline data.

The first participant was enrolled in December 2013. Follow-up assessments were conducted from January 2014 to February 2017.

### Intervention and Comparator Delivery

Study personnel delivered the intervention to participants in the intervention arm during the training session. During this session, participants received the login credentials for their accounts, logged in to the app, and reviewed its features as part of the formal training session. Participants were instructed to begin logging their BG levels on the same day and to log in regularly to review their records and emerging glycemic patterns. Within the first weeks of the study, coordinators received requests from participants for additional technology training. In response, participants were offered weekly technology-training appointments with study coordinators during the first 4 weeks of engagement. There was no equivalent training session for participants in the control group.

### Baseline Characteristics and Comparison

The sample was predominantly female (147/219, 67.1%) and diverse in age, with a mean age of 51.7 (range 23-66) years. The majority of participants were Hispanic (112/219, 51.1%) or African American (92/219, 42%), and a minority were born in the United States (98/219, 44.7%). More than two-thirds of participants (139/219, 63.5%) had not completed high school, fewer than half (92/219, 42%) were employed at the time of the study, and more than half (113/219, 51.6%) reported an annual income of less than US $10,000. The majority of participants (188/219, 85.8%) received health insurance through Medicare or Medicaid or were uninsured. On average, participants had poor glycemic control, with a mean HbA_1c_ of 9.89% (85 mmol/mol; 95% CI 9.62%-10.16%). Digital literacy was not assessed at baseline; however, many participants exhibited low literacy and required training in basic technology use, including use of a computer mouse [48]. Table 2 presents baseline demographic and clinical characteristics for participants included in the final analysis, including demographics associated with digital divide issues, such as age, primary language, educational attainment, employment, insurance status, and annual income.

**Table 2:** Demographic characteristics of the sample for the cluster-randomized clinical trial of MoDDa, a mobile health intervention for facilitating problem-solving in self-management of type 2 diabetes. The control arm received standard diabetes education and usual care; the intervention arm also used MoDD for up to 1 year. The trial was conducted with economically disadvantaged communities in the New York Metropolitan Area. The study was conducted between 2013 and 2018.

Characteristics		Total (N=219)	Intervention (n=111)	Control (n=108)
Age, mean (range)		52 (23-66)	51 (23 to 66)	52 (25 to 65)
Gender (female), n (%)		147 (67.1)	78 (70.3)	69 (63.9)
**Race/ethnicity, n (%)**				
	African American	92 (42.0)	52 (46.8)	40 (37.0)
	Hispanic	112 (51.1)	53 (47.7)	59 (54.6)
	Non-Hispanic White	9 (4.1)	4 (3.6)	5 (4.6)
	Asian, mixed, or other	116 (53)	55 (49.5)	61 (56.5)
	Born in the United States	98 (44.7)	61 (55.0)	37 (34.3)
**Primary language, n (%)**				
	English	115 (52.5)	63 (56.8)	52 (48.1)
	Spanish	54 (24.7)	28 (25.2)	26 (24.1)
	Bilingual	40 (18.3)	18 (16.2)	22 (20.4)
**Educational attainment, n (%)**				
	None to grade 11	139 (63.5)	65 (58.6)	74 (68.5)
	High school or more	78 (35.6)	46 (41.4)	32 (29.6)
Employed, n (%)		92 (42)	56 (50.5)	36 (33.3)
Medicare/Medicaid/uninsured, n (%)		188 (85.8)	91 (82.0)	97 (89.8)
Annual income (US $) <10,000, n (%)		113 (51.6)	67 (60.4)	46 (42.6)
Married, n (%)		99 (45.2)	45 (40.5)	54 (50.0)
Depressive symptoms (2-item Patient Health Questionnaire), n (%)		47 (21.5)	32 (28.8)	15 (13.9)

^a^MoDD: Mobile Diabetes Detective.

### Characterizing Usage

Analysis of usage logs showed that, of the 111 participants who received the intervention, 44 (39.6%) engaged with MoDD at least once per week during the first 30 days, and 22 (19.8%) continued weekly engagement through 90 days. Over the course of the 1-year study, participants in the intervention arm logged in an average of 29 times (range 0-759 times), recorded an average of 246 BG readings through SMS text messaging or the MoDD web interface (range 0-3949 BG readings), and set an average of 4 goals during the study (range 0-29 goals). These measures varied substantially across participants (see Figure S2 in Multimedia Appendix 1).

### Outcomes

The primary analysis followed the intent-to-treat principle; all participants with baseline measures were included in the analysis.

### Descriptive Statistics Per Clusters

Eight sites were enrolled in the trial and randomized to the intervention arm (n=4, median number of participants per site 31; range 30-34) or the control arm (n=4, median number of participants per site 30.5; range 30-31).

The breakdown of outcomes by FQHC cluster is provided in Table S1 and Figure S2 in Multimedia Appendix 1. Overall, there was high variability in both the direction and magnitude of change across clusters. This was particularly evident among FQHCs in the intervention arm. Whereas FQHCs in the control arm showed a relatively consistent trend in HbA_1c_ change over time, 2 FQHCs in the intervention arm showed no change in HbA_1c_.

### Differences in Outcomes Between Groups

Analysis of *between-group differences in primary outcomes* showed no statistically significant differences between arms for HbA_1c_ or DPSI (Table 3). There was a statistically significant difference in SDSCA diet-specific scores at 12 months (intervention: mean 4.48 days, 95% CI 4.14-4.81 days; control: mean 5.17 days, 95% CI 4.84-5.49 days; difference=0.69 days; *P*=.004), with the control arm showing better diet management than the intervention arm.

Analysis of *between-group differences in secondary outcomes* showed no significant differences for either outcome at either follow-up (see Table 3).

**Table 3:** Analysis of differences in the primary outcomes (HbA1ca, problem-solving in diabetes [DPSIb], and diabetes self-care [SDSCAc]) for the cluster-randomized clinical trial of MoDDd, a mobile health intervention for facilitating problem-solving in self-management of type 2 diabetes. The control arm received standard diabetes education and care; the intervention arm also used MoDD for up to 1 year. The trial was conducted with economically disadvantaged communities in the New York Metropolitan Area. The study was conducted between 2013 and 2018. A higher mean score indicates a more positive value in the outcome for DPSI, DSESe, and all subscales of SDSCA. A lower score indicates a more positive value in the outcomes for PAIDf. Mean scores (and corresponding 95% CIs) are model-based estimates.

Outcome				Intervention, mean (95% CI)		Control, mean (95% CI)		*P* value
**HbA_1c_**								
	Baseline			9.81 (9.42-10.2)		9.95 (9.55-10.34)		.63
	4-week follow-up			9.29 (8.05-10.07)		9.51 (8.81-10.21)		.67
	3-month follow-up			9.23 (8.76-9.7)		8.92 (8.46-9.38)		.35
	12-month follow-up			9.36 (8.95-9.78)		9.58 (9.15-10.01)		.47
**DPSI**								
	Baseline			3.89 (3.56-4.2)		3.84 (3.52-4.16)		.75
	4-week follow-up			3.91 (3.58-4.24)		3.9 (3.57-4.23)		.95
	3-month follow-up			3.85 (3.53-4.18)		3.97 (3.64-4.29)		.44
	12-month follow-up			3.9 (3.57-4.23)		3.75 (3.42-4.07)		.29
**PAID**								
	Baseline			40.52 (35.28-45.77)		34.1 (28.78-39.43)		.09
	4-week follow-up			35.67 (30.07-41.27)		29.51 (23.97-35.04)		.12
	3-month follow-up			34.7 (29.2-40.2)		27.98 (22.47-33.49)		.09
	12-month follow-up			30.93 (25.42-36.44)		29.78 (24.35-35.20)		.77
**DSES**								
	Baseline			6.95 (6.63-7.27)		7.11 (6.78-7.43)		.50
	4-week follow-up			7.3 (6.95-7.66)		7.57 (7.23-7.92)		.29
	3-month follow-up			7.53 (7.19-7.88)		7.83 (7.48-8.17)		.23
	12-month follow-up			7.4 (7.05-7.75)		7.8 (7.47-8.14)		.10
**SDSCA**								
	**SDSCA general diet**							
		Baseline	4.09 (3.62-4.56)		4.41 (3.94-4.89)		.26	
		4-week follow-up	4.89 (4.38-5.4)		5.29 (4.79-5.79)		.20	
		3-month follow-up	4.86 (4.37-5.36)		5.16 (4.67-5.65)		.34	
		12-month follow-up	4.92 (4.42-5.42)		4.84 (4.35-5.33)		.80	
	**SDSCA specific diet**							
		Baseline	4.39 (4.09-4.69)		3.97 (3.67-4.27)		.05	
		4-week follow-up	4.85 (4.5-5.19)		4.9 (4.57-5.24)		.82	
		3-month follow-up	4.84 (4.51-5.17)		4.67 (4.34-5.00)		.48	
		12-month follow-up	4.48 (4.14-4.81)		5.17 (4.84-5.49)		.004^g^	
	**SDSCA exercise**							
		Baseline	3.17 (2.6-3.74)		3.14 (2.57-3.71)		.94	
		4-week follow-up	4.09 (3.49-4.7)		3.48 (2.88-4.08)		.16	
		3-month follow-up	3.66 (3.06-4.25)		3.83 (3.24-4.43)		.68	
		12-month follow-up	3.72 (3.12-4.31)		3.6 (3.02-4.19)		.79	
	**SDSCA blood glucose tracking**							
		Baseline	3.88 (3.33-4.43)		4.43 (3.88-4.98)		.17	
		4-week follow-up	5.05 (4.46-5.65)		4.8 (4.22-5.39)		.55	
		3-month follow-up	4.77 (4.19-5.35)		4.88 (4.3-5.46)		.79	
		12-month follow-up	4.39 (3.81-4.98)		4.22 (3.65-4.79)		.67	
	**SDSCA foot care**							
		Baseline	5.01 (4.39-5.63)		5.28 (4.65-5.9)		.45	
		4-week follow-up	5.63 (4.97-6.3)		5.42 (4.76-6.07)		.59	
		3-month follow-up	5.79 (5.14-6.44)		5.48 (4.83-6.13)		.42	
		12-month follow-up	6.26 (5.6-6.9)		6.09 (5.45-6.73)		.67	

^a^HbA_1c_: glycated hemoglobin.

^b^DPSI: Diabetes Problem-Solving Inventory.

^c^SDSCA: Summary of Diabetes Self-Care Activities.

^d^MoDD: Mobile Diabetes Detective.

^e^DSES: Diabetes Self-Efficacy Scale.

^f^PAID: Problem Areas in Diabetes.

^g^Statistical significance.

### Longitudinal Trends in Outcomes

Analysis of *within-group changes in primary outcomes across study periods* for the intervention and control arms showed the following (Table 4).

For *HbA_1c_,* there was no change at 4 weeks in either arm. Both arms showed reductions in HbA_1c_ from baseline to 3 months (intervention: –0.58%, *P*=.005; control: –1.03%, *P*<.001). However, from baseline to 12 months, only the intervention arm showed a significant reduction in HbA_1c_ (intervention: –0.45%, *P*=.01; control: –0.37%, *P*=.047, not significant after adjustment for multiple comparisons).

For *problem-solving abilities (DPSI),* there was no change in either arm at any follow-up time point.

For *diabetes self-care activities (SDSCA),* the results were mixed across subscales. For *general diet*, both arms improved at 4 weeks and 3 months, but only the intervention arm improved from baseline to 12 months. For *specific diet,* the control arm improved across all 3 periods, whereas the intervention arm improved only at 3 months. For *exercise,* the intervention arm improved at 4 weeks and 12 months, whereas the control arm improved at 3 months. For *frequency of BG testing,* only the intervention arm showed improvement at 4 weeks and 3 months. For *diabetes foot care,* the intervention arm showed improvement at 3 months, and both arms showed improvement at 12 months.

Analysis of within-arm changes in *secondary outcomes* showed improvement in PAID in the intervention arm at 3 months and 12 months. For diabetes self-efficacy (DSES), the control arm improved at 4 weeks, and both arms improved at 3 and 12 months.

**Table 4:** Change in outcomes across study periods for each of the arms for the cluster-randomized clinical trial of MoDDa, a mobile health intervention for facilitating problem-solving in self-management of type 2 diabetes. The control arm received standard diabetes education and care; the intervention arm also used MoDD for up to 1 year. The trial was conducted with economically disadvantaged communities in the New York Metropolitan Area. The study was conducted between 2013 and 2018.b

Outcomes				Intervention										Control				
				Mean/mean change (95% CI)				*P* value						Mean/mean change (95% CI)		*P* value		
**Primary outcomes**																		
	**HbA_1c_^c^ (%)**																	
		Baseline			9.81 (9.42 to 10.2)				N/A^d^		9.95 (9.55 to 10.34)						N/A	
		HbA_1c_ change: baseline to 4 weeks			–0.53 (–1.0 to 0.0)				.17		–0.44 (–1.0 to 0.0)						.20	
		HbA_1c_ change: baseline to 3 months			–0.58 (–1.08 to –0.08)				.005^e^		–1.03 (–1.52 to –0.55)						<.001^e^	
		HbA_1c_ change: baseline to 12 months			–0.45 (–0.86 to –0.04)				.01^e^		–0.37 (–0.81 to –0.08)						.047^f^	
	**DPSI^g^**																	
		Baseline			3.89 (3.56 to 4.2)				N/A		3.84 (3.52 to 4.16)						N/A	
		DPSI change: baseline to 4 weeks			0.02 (–0.1 to 0.1)				.70		0.06 (0.0 to 0.1)						.32	
		DPSI change: baseline to 3 months			–0.03 (–0.18 to 0.12)				.61		0.13 (–0.02 to 0.26)						.04	
		DPSI change: baseline to 12 months			0.01 (–0.12 to 0.16)				.81		–0.09 (–0.22 to 0.04)						.11	
**SDSCA^h^**																		
	**SDSCA GD^i^**																	
		Baseline			4.09 (3.62 to 4.56)				N/A		4.41 (3.94 to 4.89)						N/A	
		SDSCA GD change: baseline to 4 weeks			0.8 (0.26 to 1.36)				<.001^e^		0.88 (0.34 to 1.42)						<.001^e^	
		SDSCA GD change: baseline to 3 months			0.77 (0.20 to 1.34)				<.001^e^		0.74 (0.20 to 1.28)						<.001^e^	
		SDSCA GD change: baseline to 12 months			0.83 (0.30 to 1.36)				<.001^e^		0.43 (–0.08 to 0.94)						.05	
	**SDSCA SD^j^**																	
		Baseline			4.39 (4.09 to 4.69)				N/A		3.97 (3.67 to 4.27)						N/A	
		SDSCA SD change: baseline to 4 weeks			0.46 (–0.02 to 0.94)				.02		0.94 (0.45 to 1.43)						<.001^e^	
		SDSCA SD change: baseline to 3 months			0.45 (–0.02 to 0.93)				.03		0.7 (0.20 to 1.21)						.001^e^	
		SDSCA SD change: baseline to 12 months			0.1 (–0.38 to 0.55)				.64		1.2 (0.70 to 1.70)						<.001^e^	
	**SDSCA E^k^**																	
		Baseline			3.17 (2.6 to 3.74)				N/A		3.14 (2.57 to 3.71)						N/A	
		SDSCA E change: baseline to 4 weeks			0.92 (0.34 to 1.50)				<.001^e^		0.34 (–0.28 to 0.96)						.18	
		SDSCA E change: baseline to 3 months			0.48 (–0.04 to 1.02)				.03		0.69 (0.14 to 1.24)						.003^e^	
		SDSCA E change: baseline to 12 months			0.55 (–0.03 to 1.11)				.02^e^		0.46 (–0.11 to 1.04)						.05	
	**SDSCA BG^l^ tracking**																	
		Baseline			3.88 (3.33 to 4.43)				N/A		4.43 (3.88 to 4.98)						N/A	
		SDSCA BG change: baseline to 4 weeks			1.17 (0.51 to 1.4)				<.001^e^		0.37 (–0.31 to 1.05)						.19	
		SDSCA BG change: baseline to 3 months			0.89 (0.23 to 1.55)				.002^e^		0.45 (–0.20 to 1.10)						.10	
		SDSCA BG change: baseline to 12 months			0.52 (–0.13 to 1.16)				.05		–0.21 (–0.81 to 0.39)						.40	
	**SDSCA FC^m^**																	
		Baseline			5.01 (4.39 to 5.63)				N/A		5.28 (4.65 to 5.9)						N/A	
		SDSCA FC change: baseline to 4 weeks			0.63 (–0.02 to 1.28)				.02		0.14 (–0.49 to 0.66)						.77	
		SDSCA FC change: baseline to 3 months			0.78 (0.12 to 1.45)				.005^e^		0.2 (–0.43 to 0.82)						.45	
		SDSCA FC change: baseline to 12 months			1.23 (0.58 to 1.92)				<.001^e^		0.81 (0.14 to 1.48)						.005^e^	
**Secondary outcomes**																		
	**PAID^n^**																	
		Baseline	40.52 (35.28 to 45.77)				N/A						34.1 (28.78 to 39.43)		N/A			
		PAID change: baseline to 4 weeks	4.85 (–0.19 to 9.89)				.02						4.6 (–0.3 to 9.49)		.02			
		PAID change: baseline to 3 months	5.82 (0.95 to 10.7)				.004^e^						6.13 (1.27 to 10.97)		.03			
		PAID change: baseline to 12 months	9.59 (4.71 to 14.48)				<.001^e^						4.33 (–0.4 to 9.05)		.03			
	**DSES^o^**																	
		Baseline	6.95 (6.63 to 7.27)				N/A						7.11 (6.78 to 7.43)		N/A			
		DSES change: baseline to 4 weeks	0.36 (–0.02 to 0.75)				.02						0.47 (0.09 to 0.85)		.003^e^			
		DSES change: baseline to 3 months	0.59 (0.21 to 0.96)				<.001^e^						0.72 (0.35 to 1.1)		<.001^e^			
		DSES change: baseline to 12 months	0.45 (0.08 to 0.83)				.004^e^						0.7 (0.33 to 1.07)		<.001^e^			

^a^MoDD: Mobile Diabetes Detective.

^b^A positive value indicates an increase.

^c^HbA_1c_: glycated hemoglobin.

^d^N/A: not applicable.

^e^Statistical significance.

^f^Lack of statistical significance after adjusting for multiple comparisons (critical values of *P*=.02). For the 5 SDSCA subscales, all *P* values were false discovery rate–adjusted.

^g^DPSI: Diabetes Problem-Solving Inventory.

^h^SDSCA: Summary of Diabetes Self-Care Activities.

^i^GD: general diet.

^j^SD: specific diet.

^k^E: exercise.

^l^BG: blood glucose.

^m^FC: foot care.

^n^PAID: Problem Areas in Diabetes.

^o^DSES: Diabetes Self-Efficacy Scale.

### Dosage Effect Analysis

Overall, there was no association between MoDD usage and HbA_1c_ within the intervention arm. The level of engagement was not associated with the change in HbA_1c_ from baseline to 12 months (*P*=.26 for logins and *P*=.70 for BG readings). Notably, we did not examine trends in HbA_1c_ from baseline to 12 months by engagement strata because of data limitations, including a large percentage of missing values and a limited sample size. Furthermore, to examine the potential association between distress and engagement, we included a distress measure (PAID) in the model as a confounder. The differences between adjusted and unadjusted (current) HbA_1c_ estimates were generally small, ranging from 0.01% to 0.29% (see Multimedia Appendix 1). Finally, we conducted a sensitivity analysis excluding observations in the intervention arm with zero engagement. We then estimated HbA_1c_ change across the 2 arms. There was no difference in the estimates, likely because the number of observations with zero engagement in the intervention arm was very small.

### Qualitative Feedback From Participants

Analysis of qualitative interviews with study participants revealed that participants were able to understand and follow the steps of the problem-solving process and found the process intuitive. Using MoDD helped participants recognize problematic BG patterns, of which many had previously been unaware. Furthermore, the extensive collection of possible behavioral triggers helped participants reflect on their own behaviors and identify the most plausible triggers. Participants also found sufficient variety in the action-oriented goals to select goals consistent with their values and preferences. Finally, receiving feedback from MoDD on changes in BG levels provided additional motivation to engage in problem-solving and improve BG management. More details on the qualitative results are available elsewhere [51].

### Harms

No intervention-related adverse events, privacy breaches, or technical failures were reported during the trial.

## Discussion

### Principal Findings

This cluster-randomized controlled trial evaluated the efficacy of MoDD, a fully automated mHealth intervention designed to support diabetes problem-solving. The primary outcome of the study was improvement in HbA_1c_ from baseline to 12 months in the intervention arm, which received both standard diabetes education and access to MoDD for 4 weeks and up to 1 year, compared with the control group, which only received diabetes education and standard care at their FQHC. Additional outcomes were improvement in problem-solving abilities (DPSI), diabetes self-management behaviors (SDSCA), diabetes self-efficacy (DSES), and diabetes distress (PAID). Contrary to our primary hypothesis, we found no statistically significant difference in HbA_1c_ between the intervention and control groups at 12 months. However, participants in the intervention arm demonstrated sustained within-group improvement in HbA_1c_ over time, whereas participants in the control group did not. There was no change in problem-solving abilities (DPSI) in either study arm at any time point. There was a significant increase in the frequency of BG monitoring (SDSCA) in the intervention arm at 3 months; however, this increase was not sustained at 12 months. The remaining self-care activities improved inconsistently across study arms and periods. Notably, participants in both arms reported moderate to high diabetes distress (PAID) at baseline. Furthermore, participants in the intervention arm scored above the clinically established threshold for high diabetes distress (>40) [60], also scored high on depression measures, and reported increases in diabetes distress across all study periods, whereas no such increase was observed in the control arm.

These findings differ somewhat from those of previous studies examining mHealth interventions for diabetes self-management and problem-solving. On the one hand, the improvement in HbA_1c_ in the intervention arm is consistent with previous reports of computer-based interventions for both diabetes self-management [29,61] and expert-moderated problem-solving [11]. Although the effect size of the improvement in HbA_1c_ at 12 months after baseline was relatively modest (–0.45%, 95% CI –0.86% to –0.04%), it was larger than the pooled decrease in HbA_1c_ reported in a meta-analysis of health information technology interventions for medically underserved adults with diabetes (–0.27%, 95% CI –0.49% to –0.04%) [29]. However, the comparable improvement in HbA_1c_ in the control arm was somewhat unexpected and may have been due to the Hawthorne effect [62], regression to the mean [63], or the impact of usual care [64]. Nevertheless, the decrease in HbA_1c_ in the control arm was not sustained at 12 months, highlighting the potential benefit of MoDD for achieving sustained HbA_1c_ improvement. The high compliance rate with the 12-month follow-up in both arms further strengthens the robustness of this finding.

The lack of change in problem-solving abilities is also inconsistent with prior studies [65]. However, this may be due to differences in the definition and operationalization of problem-solving underlying DPSI and the definition used to guide the design of MoDD. Whereas DPSI primarily focuses on individuals’ *ability to overcome specific barriers* to self-management, MoDD was primarily designed to support individuals in *reasoning and discovery* while problem-solving around high BG levels. This finding points to the need for validated assessment tools that focus on these aspects of diabetes problem-solving. Furthermore, improved problem-solving may not necessarily translate into behavior change, as social determinants of health-related barriers, such as food insecurity, transportation challenges, and housing instability, may be difficult for individuals living in low-resource communities to overcome, thereby limiting their ability to address these barriers effectively [66].

The increase in the frequency of BG testing is not surprising, given that MoDD specifically encouraged individuals to frequently check their BG levels. However, the lack of a sustained increase at 12 months points to barriers to continued BG monitoring among individuals with type 2 diabetes, including high cost, inconvenience, and discomfort [44]. Self-monitoring data are critical for the development of personalized interventions in chronic disease self-management, and the lack of such data can limit the effectiveness of implementing these interventions. Increased adoption of continuous glucose monitoring may alleviate some of these barriers; however, continuous glucose monitoring remains rarely available to individuals with type 2 diabetes, particularly those without health insurance and others in medically underserved communities [67]. More broadly, inconsistent changes in reported self-care behaviors highlight the challenges of both achieving sustained behavior change and reliably measuring such change through self-reports. Technologies for self-monitoring of meals, physical activity, and medication adherence may help provide more objective assessments of changes in these behaviors. However, barriers to adoption of these technologies remain among medically underserved communities, which also experience low digital and health literacy [44].

The sustained increase in diabetes distress in the intervention arm, although concerning, is not surprising and may be attributed to increased awareness of high BG levels among participants in the intervention arm, who reported a higher frequency of BG monitoring [68]. Arguably, such awareness is a necessary precondition for behavior change and may help individuals progress through the stages of behavior change, for example, from precontemplation to contemplation and action [50]. However, the potential positive effects of this increased awareness must be balanced against the possibility of exacerbating diabetes distress and burnout [68].

### Implications for Future Research and Technological Innovation

This study highlighted several open questions that suggest directions for future research. For example, it suggested that low digital health literacy and limited experience with even simple technologies may have prevented participants from fully benefiting from mHealth interventions for problem-solving, such as MoDD. In fact, many participants in this study lacked basic technology literacy and required additional technology training and support [48]. These trends may present challenges not only for efficacy assessment in clinical trials, but also for the feasibility, sustainability, and scalability of broader MoDD deployment. At the same time, previous research has highlighted the potential for technological interventions to reduce health disparities [69]. For example, widely adopted, inexpensive, and scalable technologies, such as SMS text messaging, can lower barriers to engagement with health interventions among diverse communities and have demonstrated efficacy in prior research [70]. Furthermore, conversational agents and chatbots, particularly those powered by artificial intelligence and large language models, have gained considerable prominence as mechanisms for engaging individuals from diverse communities in health management [71]. Given their reliance on conversation as the primary interaction mechanism, these technologies may be particularly well suited to supporting cognitive skills such as problem-solving, which require deliberation and consideration of alternatives. The problem-solving framework that guided the design of MoDD, and its translation into a stepwise problem-solving process, may provide a useful blueprint for structuring problem-solving conversations with artificial intelligence–powered chatbots.

### Limitations

This study has several limitations. First, although the final sample size included in the analysis exceeded the sample size required to achieve 90% power, it may still have been insufficient to detect more subtle changes in outcomes. Second, the relatively high refusal rate may have introduced selection bias. Third, reliance on model estimates for the 3- and 12-month HbA_1c_ outcomes to account for missing and misaligned data points from routine clinical data extracted from the electronic health record may have introduced bias. To address this issue, we used linear mixed models, which are particularly well-suited to providing unbiased estimates in the presence of missing data. The behavioral measures used in the study relied on participant self-assessments and may have been subject to bias, particularly when administered through interviews with research coordinators. Declining engagement with the intervention over time is consistent with patterns observed in many digital health interventions and may have reduced the magnitude of the clinical effects. Finally, the study did not track adherence to diabetes medication, including insulin use, which is a critical factor in successful self-management.

### Conclusions

Overall, this study contributes new evidence regarding the benefits of mHealth for problem-solving and highlights both its potential positive effects on BG management and the challenges of achieving sustained improvement. MoDD is innovative because, unlike prior interventions, it operates autonomously and tailors support to individuals’ self-monitoring data. Despite the widespread proliferation of mHealth interventions supporting diabetes self-management, only a few have focused on promoting problem-solving, which provides a critical cognitive foundation for successful self-management [1-3]. Although engagement with MoDD did not result in measurable improvement in problem-solving skills, at least as assessed by DPSI, the qualitative study accompanying the trial found that participants did engage in problem-solving, found the problem-solving stages intuitive, and made multiple discoveries while using MoDD [51]. The intervention relied on basic mobile phone access and SMS text messaging rather than smartphones, suggesting its feasibility in settings with limited digital infrastructure. Given the design of the intervention and the demographic characteristics of the study sample, we expect these results to generalize to similar mHealth interventions implemented in economically disadvantaged, medically underserved, and ethnically and racially diverse populations. If implemented in routine clinical practice, MoDD could complement traditional diabetes education and help improve glycemic control. However, additional resources from health centers would likely be required to provide the training and technology support needed for successful engagement.

## Appendix

Multimedia Appendix 1 Additional analysis.

Multimedia Appendix 2 Mobile Diabetes Detective CONSORT (Consolidated Standards of Reporting Trials) checklist.

Multimedia Appendix 3 CONSORT-EHEALTH checklist. CONSORT: Consolidated Standards of Reporting Trials.

## Abbreviations

BG: blood glucose
CDN: Clinical Directors Network
CONSORT: Consolidated Standards of Reporting Trials
DPSI: Diabetes Problem-Solving Inventory
DSES: Diabetes Self-Efficacy Scale
DSMES: diabetes self-management education and support
FQHC: Federally Qualified Health Center
HbA1c: glycated hemoglobin
mHealth: mobile health
MoDD: Mobile Diabetes Detective
PAID: Problem Areas in Diabetes
SDSCA: Summary of Diabetes Self-Care Activities
SMD: standardized mean difference

## Data Availability

Given the sensitive nature of the data collected in this study, the datasets used and analyzed are available from the corresponding author (LM) to qualified requesters upon reasonable request.
