# Supplementary material for: Promoting Problem-Solving Among Low-Income Adults With Type 2 Diabetes: Cluster-Randomized Controlled Trial of a Mobile Health Intervention With SMS Text Messaging (Mobile Diabetes Detective)
Source: J Med Internet Res. 2026 Jul 13;28:e82305. doi: 10.2196/82305 (PMC13408465; doi:10.2196/82305)
Supplement: Multimedia Appendix 1 [file jmir_v28i1e82305_app1.docx]

1. **MoDD design**

**Example of a behavioral trigger: “**I occasionally skip breakfast”

**Example of an educational segment:** “It is important for people with diabetes to eat regular meals to maintain consistent blood sugar levels. When you skip breakfast, your body is forced to make the fuel you need for energy from other sources. Over time, your body can become less effective in using insulin, and become insulin resistant. Eating a healthy breakfast, and regular meals every 4 hours can help keep your insulin level and blood sugar levels more stable.”

**Example of a motivational message:** “Think about how good you might feel if you make a few small changes to your routine every day.”

1. **MoDD usage statistics**

Figure 1: MoDD usage statistics: top: number of recorded BG levels (in bins of 25); middle: number of logins (in bins of 10), bottom: number of goals set in the study.

1. **Descriptive statistics per cluster**

| Intervention (n=111) | Site 1 (n=29)  Mean (95% CI) | Site 2 (n=26)  Mean (95% CI) | Site 3 (n=28)  Mean (95% CI) | Site 4 (n=28)  Mean (95% CI) |
| --- | --- | --- | --- | --- |
| Baseline | 9.74 (9.2; 10.28) | 9.32 (8.83; 9.82) | 9.97 (9.26; 10.68) | 10.19 (9.35; 11.04) |
| 3m follow-up | 9.57 (8.87; 10.27) | 8.30 (7.67; 8.93) | 9.77 (8.86; 10.68) | 9.35 (8.25; 10.44) |
| 12m follow-up | 9.6 (9.03; 10.16) | 8.44 (7.93; 8.96) | 10.38 (9.54; 11.22) | 9.16 (8.25; 10.07) |

| Control (n=107) | Site 5 (n=28)  Mean (95% CI) | Site 6 (n=30)  Mean (95% CI) | Site 7 (n=25)  Mean (95% CI) | Site 8 (n=24)  Mean (95% CI) |
| --- | --- | --- | --- | --- |
| Baseline | 9.77 (9.12; 10.42) | 9.8 (9.22; 10.37) | 9.83 (9.03; 10.64) | 10.46 (9.82; 11.1) |
| 3m follow-up | 8.73 (7.92; 9.54) | 9.09 (8.42; 9.75) | 9.08 (8.11; 10.04) | 8.41 (7.54; 9.28) |
| 12m follow-up | 9.37 (8.69; 10.05) | 9.87 (9.22;10.52) | 9.69 (8.8; 10.58) | 9.24 (8.46; 10.02) |

Table 1: HbA1c measures per cluster; Control group (mean scores (and corresponding SEs) are model-based estimates).

Figure 2: Change in HbA1c per cluster (left: Intervention Arm, right: Control Arm)

1. **Analysis of the distress measure (PAID) as a potential confounder**

We included distress measure (PAID) in the model as a confounder. The difference between adjusted and unadjusted (current) estimates was mostly small.

|  | Confidence interval adjusted for multiple tests | | | | | |
| --- | --- | --- | --- | --- | --- | --- |
|  | Intervention | | | Control | | |
| HbA1c ,%, | Current unadjusted | adjusted | SMD | Current unadjusted | adjusted | SMD |
| Unadjusted results |  |  |  |  |  |  |
| Baseline to 5w | -0.53 | -0.52 | 0.02 | -0.44 | -0.73 | 0.69 |
| Baseline to 3m | -0.58 | -0.60 | 0.10 | -1.03 | -1.04 | 0.04 |
| Baseline to 12m | -0.45 | -0.47 | 0.10 | -0.37 | -0.42 | 0.24 |

Table 2: Analysis of the distress measure (PAID) as a potential confounder in the model

1. **Analysis of missing values**

We calculated percent with missing values by time period and selected baseline variables. There was no clear evidence of bias in missing according to selected baseline variables.

| Period | INT | Missing |
| --- | --- | --- |
| BL | Int | 0.00% |
| BL | Control | 1.90% |
| 4wk | Int | 87.40% |
| 4wk | Control | 83.30% |
| 3mo | Int | 49.60% |
| 3mo | Control | 45.40% |
| 12mo | Int | 26.10% |
| 12mo | Control | 30.60% |

Table 3: Proportion of missing values at different milestones. The lowest is at 12 months, the primary endpoint of the trial.

| Period | Age | Missing |
| --- | --- | --- |
| BL | <54 | 0.00% |
| BL | ≥54 | 0.90% |
| 4wk | <54 | 85.60% |
| 4wk | ≥54 | 84.70% |
| 3mo | <54 | 46.20% |
| 3mo | ≥54 | 46.80% |
| 12mo | <54 | 28.80% |
| 12mo | ≥54 | 25.20% |

Table 4: Proportion of missing values at different milestones by age (below 54, at or above 54). At each milestone, there are no notable differences in the proportion of missing values for different age groups.

| Period | Gender | Missing |
| --- | --- | --- |
| BL | F | 0.00% |
| BL | M | 1.40% |
| 4wk | F | 86.20% |
| 4wk | M | 83.10% |
| 3mo | F | 49.00% |
| 3mo | M | 42.30% |
| 12mo | F | 24.80% |
| 12mo | M | 32.40% |

Table 5: Proportion of missing values at different milestones by sex (male, female). At each milestone, there are no notable differences in the proportion of missing values for different sexes.

| Period | Edu | Missing |
| --- | --- | --- |
| BL | <HS | 1.20% |
| BL | ≥HS | 0.00% |
| 4wk | <HS | 85.90% |
| 4wk | ≥HS | 84.70% |
| 3mo | <HS | 47.10% |
| 3mo | ≥HS | 46.60% |
| 12mo | <HS | 25.90% |
| 12mo | ≥HS | 28.20% |

Table 6: Proportion of missing values at different milestones by education (below high school, at or above high school). At each milestone, there are no notable differences in the proportion of missing values for different educational levels.

| Period | PHQ2 | Missing |
| --- | --- | --- |
| BL | <3 | 0.60% |
| BL | ≥3 | 0.00% |
| 4wk | <3 | 85.10% |
| 4wk | ≥3 | 85.10% |
| 3mo | <3 | 43.50% |
| 3mo | ≥3 | 57.40% |
| 12mo | <3 | 26.80% |
| 12mo | ≥3 | 23.40% |

Table 7: Proportion of missing values at different milestones by different levels of depression as measure by PHQ2 (below 3 is normal, at or above 3 is high). At each milestone, there are no notable differences in the proportion of missing values for baseline, 4 weeks, and 12 months (primary end-point). There was a moderate difference in the proportion of missing values for different levels of depression at 3months.

| INT | Period | Actual N | % of Missing | # of Missing |
| --- | --- | --- | --- | --- |
| 1 | 1 | 111 | 0% | 0 |
| 2 | 1 | 107 | 0% | 0 |
| 1 | 2 | 14 | 87% | 97 |
| 2 | 2 | 18 | 83% | 90 |
| 1 | 3 | 56 | 50% | 55 |
| 2 | 3 | 59 | 45% | 49 |
| 1 | 4 | 82 | 26% | 29 |
| 2 | 4 | 75 | 31% | 33 |

Table 8: Missing values by arm and time period (1: baseline, 2: 4 weeks, 3: 3months, and 4: 12 months).

We also estimated the main outcome (HbA1c) using two methods to handle missing values

(1) Full information Maximum likelihood method (FIML) and

(2) Multiple imputation method (MI).

We compared the difference in estimates of HbA1c change from period 1 to periods 2, 3, and 4 based on these two methods and estimates based on the current method (remove data with missing value).

We used standardized mean difference (SMD) to measure the difference of FIML and MI estimates vs. current estimates. The SMDs between FIML and current method were less than 0.13 for all estimates. The SMDs between MI and current method were all less than 0.5. Therefore, the impact of missing values on the bias of effect of intervention estimates was small.

|  |  | HbA1c change from period 1 | | | | |
| --- | --- | --- | --- | --- | --- | --- |
|  |  |  | FIML vs Current | | MI vs Current | |
| INT | period | Current | FIML | SMD | MI | SMD |
| 1 | 2 | -0.53 | -0.53 | 0.01 | -0.54 | 0.04 |
| 1 | 3 | -0.58 | -0.59 | 0.03 | -0.67 | 0.35 |
| 1 | 4 | -0.45 | -0.45 | 0.00 | -0.40 | 0.23 |
| 2 | 2 | -0.44 | -0.42 | 0.04 | -0.56 | 0.37 |
| 2 | 3 | -1.03 | -1.01 | 0.05 | -0.95 | 0.36 |
| 2 | 4 | -0.37 | -0.33 | 0.12 | -0.47 | 0.47 |

Table 9: Analysis of the impact of missing values on the primary outcome conducted with FIML, MI, and the current method.

Note: SMD <0.2 was considered as no differences; SMD = 0.2, small difference; SMD = 0.5, medium difference; and SMD = 0.8, large difference.

1. **Analysis of change within each group**

Figure 3: Change in HbA1c for the two study conditions. Mean scores (and corresponding SEs) are model-based estimates.

1. **Analysis of change between study arms**

| **Outcome** | **Intervention** | **Control** |  |
| --- | --- | --- | --- |
| **HbA1c** | **Mean (95% CE)** | **Mean (95% CE)** | **p-value** |
| Baseline | 9.81 (9.42; 10.2) | 9.95 (9.55; 10.34) | 0.63 |
| 5-week follow-up | 9.29 (8.05; 10.07) | 9.51 (8.81; 10.21) | 0.67 |
| 3-months follow-up | 9.23 (8.76; 9.7) | 8.92 (8.46; 9.38) | 0.35 |
| 12-months follow-up | 9.36 (8.95; 9.78) | 9.58 (9.15; 10.01) | 0.47 |
| **DPSI** |  |  |  |
| Baseline | 3.89 (3.56; 4.2) | 3.84 (3.52; 4.16) | 0.75 |
| 5-week follow-up | 3.91 (3.58; 4.24) | 3.9 (3.57; 4.23) | 0.95 |
| 3-months follow-up | 3.85 (3.53; 4.18) | 3.97 (3.64; 4.29) | 0.44 |
| 12-months follow-up | 3.9 (3.57; 4.23) | 3.75 (3.42; 4.07) | 0.29 |
| **PAID** |  |  |  |
| Baseline | 40.52 (35.28; 45.77) | 34.1 (28.78; 39.43) | 0.09 |
| 5-week follow-up | 35.67 (30.07; 41.27) | 29.51 (23.97; 35.04) | 0.12 |
| 3-months follow-up | 34.7 (29.2; 40.2) | 27.98 (22.47; 33.49) | 0.09 |
| 12-months follow-up | 30.93 (25.42; 36.44) | 29.78 (24.35; 35.20) | 0.77 |
| **DSES** |  |  |  |
| Baseline | 6.95 (6.63; 7.27) | 7.11 (6.78; 7.43) | 0.5 |
| 5-week follow-up | 7.3 (6.95; 7.66) | 7.57 (7.23; 7.92) | 0.29 |
| 3-months follow-up | 7.53 (7.19; 7.88) | 7.83 (7.48; 8.17) | 0.23 |
| 12-months follow-up | 7.4 (7.05; 7.75) | 7.8 (7.47; 8.14) | 0.1 |
| **SDSCA** |  |  |  |
| **SDSCA General Diet** |  |  |  |
| Baseline | 4.09 (3.62; 4.56) | 4.41 (3.94; 4.89) | 0.26 |
| 5-week follow-up | 4.89 (4.38; 5.4) | 5.29 (4.79; 5.79) | 0.2 |
| 3-months follow-up | 4.86 (4.37; 5.36) | 5.16 (4.67; 5.65) | 0.34 |
| 12-months follow-up | 4.92 (4.42; 5.42) | 4.84 (4.35; 5.33) | 0. 8 |
| **SDSCA Specific Diet** |  |  |  |
| Baseline | 4.39 (4.09; 4.69) | 3.97 (3.67; 4.27) | 0.05 |
| 5-week follow-up | 4.85 (4.5; 5.19) | 4.9 (4.57; 5.24) | 0.82 |
| 3-months follow-up | 4.84 (4.51; 5.17) | 4.67 (4.34; 5.00) | 0.48 |
| 12-months follow-up | 4.48 (4.14; 4.81) | 5.17 (4.84; 5.49) | 0.004 (*) |
| **SDSCA Exercise** |  |  |  |
| Baseline | 3.17 (2.6; 3.74) | 3.14 (2.57; 3.71) | 0.94 |
| 5-week follow-up | 4.09 (3.49; 4.7) | 3.48 (2.88; 4.08) | 0.16 |
| 3-months follow-up | 3.66 (3.06; 4.25) | 3.83 (3.24; 4.43) | 0.68 |
| 12-months follow-up | 3.72 (3.12; 4.31) | 3.6 (3.02; 4.19) | 0.79 |
| **SDSCA Blood Glucose Tracking** |  |  |  |
| Baseline | 3.88 (3.33; 4.43) | 4.43 (3.88; 4.98) | 0.17 |
| 5-week follow-up | 5.05 (4.46; 5.65) | 4.8 (4.22; 5.39) | 0.55 |
| 3-months follow-up | 4.77 (4.19; 5.35) | 4.88 (4.3; 5.46) | 0.79 |
| 12-months follow-up | 4.39 (3.81; 4.98) | 4.22 (3.65; 4.79) | 0.67 |
| **SDSCA Foot Care** |  |  |  |
| Baseline | 5.01 (4.39; 5.63) | 5.28 (4.65; 5.9) | 0.45 |
| 5-week follow-up | 5.63 (4.97; 6.3) | 5.42 (4.76; 6.07) | 0.59 |
| 3-months follow-up | 5.79 (5.14; 6.44) | 5.48 (4.83; 6.13) | 0.42 |
| 12-months follow-up | 6.26 (5.6; 6.9) | 6.09 (5.45; 6.73) | 0.67 |

Table 10: Analysis of difference between arms at each study milestone for all study outcomes. Higher mean score indicates a more positive value in the outcome for DPSI, DSES, and all sub-scales of SDSCA. Lower score indicates a more positive value in the outcomes for PAID. Mean scores (and corresponding SEs) are model-based estimates.
