# Supplementary material for: Promoting Problem-Solving Among Low-Income Adults With Type 2 Diabetes: Cluster-Randomized Controlled Trial of a Mobile Health Intervention With SMS Text Messaging (Mobile Diabetes Detective)
Source: J Med Internet Res. 2026 Jul 13;28:e82305. doi: 10.2196/82305 (PMC13408465; doi:10.2196/82305)
Supplement: Multimedia Appendix 3 [file jmir_v28i1e82305_app3.pdf]

Supplementary File: CONSORT 2025 and CONSORT-EHEALTH Checklist

Manuscript Title: Promoting Problem-Solving Among Low-Income Adults with Type 2 Diabetes: A Cluster-Randomized Controlled Trial of Mobile Diabetes Detective, an mHealth Intervention with Text Messaging

Trial Registration: NCT02021591

Reporting Standards: CONSORT 2025, CONSORT-EHEALTH (v1.6)

Title and Abstract

Item	Checklist Description	Location in Manuscript
1a	Identification as a randomized trial in the title	Title
1b	Structured abstract with trial design, methods, results, conclusions	Abstract
E1	Title identifies digital modality (mHealth/SMS)	Title
E2	Abstract describes digital intervention	Abstract Methods
E3	Abstract states level of human involvement	Abstract Methods
E4	Abstract reports engagement metrics	Abstract Results
E5	Abstract includes trial registration	Abstract

Introduction

Item	Checklist Description	Location in Manuscript

2a	Scientific background and rationale	Introduction 1.1–1.2
2b	Specific objectives or hypotheses	Introduction 1.4
E6	Rationale for digital intervention	Introduction 1.2
E7	Description of theoretical framework	Introduction 1.3

Methods – Trial Design

Item	Checklist Description	Location in Manuscript
3a	Description of trial design (cluster randomized parallel trial)	Methods 2
3b	Changes to methods after trial commencement	Methods 2.4 (none)

Methods – Participants

Item	Checklist Description	Location in Manuscript
4a	Eligibility criteria for participants	Methods 2.2
4b	Settings and locations where data were collected	Methods 2.1
E8	Technology access requirements	Methods 2.2
E9	Recruitment method	Methods 2.5
E10	Digital literacy considerations	Results 3.3 / Discussion

Methods – Intervention

Item	Checklist Description	Location in Manuscript

5	Detailed description of intervention	Methods 2.3
E11	Description of digital intervention components	Methods 2.3
E12	Mode of delivery (web + SMS)	Methods 2.3
E13	Human involvement level	Methods 2.3
E14	Training procedures	Methods 2.3.3
E15	Access instructions and use parameters	Methods 2.3.3
E16	Version stability and updates	Methods 2.3.2
E17	Digital preservation / screenshots	Methods 2.3.2 + Supplement

Methods – Outcomes

Item	Checklist Description	Location in Manuscript
6a	Defined primary and secondary outcomes	Methods 2.9
6b	Changes to trial outcomes after commencement	Methods 2.9 (none)
E18	Definition of engagement metrics	Methods 2.3.3
E19	Process outcomes related to intervention use	Results 3.4

Methods – Sample Size

Item	Checklist Description	Location in Manuscript
7a	Sample size determination	Methods 2.10
7b	Interim analyses or stopping guidelines	Not applicable

Methods – Randomization

Item	Checklist Description	Location in Manuscript
8a	Sequence generation method	Methods 2.8
8b	Type of randomization	Methods 2.8
9	Allocation concealment mechanism	Methods 2.8
10	Implementation of randomization	Methods 2.8
11a	Blinding	Methods 2.8

Methods – Statistical Analysis

Item	Checklist Description	Location in Manuscript
12a	Statistical methods for outcomes	Methods 2.11
12b	Additional analyses	Methods 2.11

Results

Item	Checklist Description	Location in Manuscript
13a	Participant numbers randomized and analyzed	Results 3.1
13b	Losses and exclusions	Results 3.1
13c	CONSORT flow diagram	Figure 2
14a	Recruitment and follow-up dates	Results 3.1
15	Baseline characteristics	Table 2
16	Numbers included in analysis	Results 3.1
17a	Outcome results for each group	Tables 3–4

17b	Effect sizes and confidence intervals	Tables 3–4
18	Ancillary analyses	Results 3.5.4
19	Harms	Results 3.5.6

Discussion

Item	Checklist Description	Location in Manuscript
20	Limitations	Discussion
21	Generalizability	Discussion
22	Interpretation consistent with results	Discussion
E20	Discussion of engagement	Discussion
E21	Implementation considerations	Discussion
E22	Digital divide considerations	Discussion

Other Information

Item	Checklist Description	Location in Manuscript
23	Trial registration	Methods / Abstract
24	Protocol access	ClinicalTrials.gov
25	Funding	Acknowledgements
